# Glyoxal measurement with a proton transfer reaction time of flight mass spectrometer (PTR‐TOF‐MS): characterization and calibration

**DOI:** 10.1002/jms.3893

**Published:** 2016-11-25

**Authors:** Christof Stönner, Bettina Derstroff, Thomas Klüpfel, John N. Crowley, Jonathan Williams

**Affiliations:** ^1^ Max Planck‐Institute for Chemistry Mainz Germany

**Keywords:** PTR‐TOF‐MS, glyoxal, fragmentation, formaldehyde, glyoxal calibration

## Abstract

We examine the potential for PTR‐TOF‐MS systems to quantitatively measure glyoxal in ambient air by characterizing the response of the instrument to a dilute glyoxal sample, calibrating the system as a function of humidity. The concentration of glyoxal in a sample air‐stream was measured with an UV absorption spectrometer in parallel to a PTR‐TOF‐MS. This calibration demonstrated that the PTR‐TOF‐MS has a relatively low sensitivity to glyoxal particularly at high humidity. Extensive fragmentation of glyoxal to formaldehyde was observed. This behaviour not only desensitizes PTR‐MS system to glyoxal; it may also pose a problem to the quantification of formaldehyde. © 2016 The Authors. Journal of Mass Spectrometry Published by John Wiley & Sons Ltd.

## Introduction

Glyoxal is the smallest α‐dicarbonyl compound, having the structure HC(O)C(O)H. It is known to be directly emitted from biomass burning^1^ but most of the global annual emission to the atmosphere occurs via the oxidation of biogenic (e.g. isoprene) and anthropogenic (e.g. acetylene) volatile organic compounds (VOCs). On a global scale the majority of atmospheric glyoxal is formed by the photo‐oxidation of biogenic isoprene, which contributes approximately 47% to the global glyoxal production of 45 Tg.^2^ Acetylene oxidation is the second largest source of glyoxal (~9 Tg). Acetylene is primarily emitted from anthropogenic sources like combustion.^3^ Further anthropogenic precursor trace gases are aromatic compounds (e.g. toluene, xylenes) which undergo ring‐opening reactions in the presence of NOx (NO and NO_2_) to yield glyoxal.^4^

Under typical daytime conditions, glyoxal has an atmospheric lifetime of approximately 2 h.^2^ It is removed from the atmosphere by photolysis, oxidation by OH and heterogeneous processes. Glyoxal can be used to delineate local and regional photochemistry because its short lifetime precludes long range transport from its biogenic and anthropogenic sources.^5^ In unpolluted regions, with low NOx mixing ratios and high amounts of VOCs glyoxal photolysis may contribute to the regeneration of HOx (HO + HO_2_) in the troposphere. These conditions occur over tropical rainforests, where high emissions of isoprene may suppress ambient OH levels if recycling mechanisms such as via glyoxal photolysis did not occur.^6^, ^7^, ^8^ Additionally, glyoxal contributes to the formation of secondary organic aerosol (SOA) and cloud droplets, which in turn affect climate and air quality.^9^

Global distributions of glyoxal have been calculated through model studies and measured with space‐borne UV–VIS and near IR spectrometers. The model simulated mixing ratios of glyoxal at ground level are highest in biomass burning affected regions, areas with strong isoprene emissions and over anthropogenically polluted regions. In these environments, the ambient mixing ratios of glyoxal typically range between 10 and 100 pptv.^2^ Global observations of glyoxal have been conducted with satellites using Scanning Imaging Absorbtion SpectroMeter for Atmospheric CartograpHY (SCIAMACHY)^10^, ^11^ and Global Ozone Monitoring Experiment (GOME)^12^ and GOME‐2^13^ instruments to retrieve global total column datasets of glyoxal. While these systems provide a good estimate for the global distribution of glyoxal, they lack in spatial and temporal resolution. Comparisons between simulated and measured glyoxal distributions show reasonable agreement over land. However, over the tropical oceans, the modelled glyoxal vertical column concentration significantly underestimates the concentrations retrieved from the satellite instrument indicating an unknown source of glyoxal.^11^

The aforementioned findings have driven experimentalists to develop many measurement systems for glyoxal so as to better understand its abundance. In addition to satellite measurements, several ground‐based optical/spectroscopic devices have been used for the detection of glyoxal. These include Differential Optical Absorption Spectroscopy (DOAS),^5^, ^14^ Broadband Cavity Enhanced Absorption Spectroscopy (BBCEAS)^15^ and Laser Induced Phosphorescence (LIP) spectroscopy.^16^ These optical devices allow the sensitive measurement of glyoxal with a time resolution up to 1 min. Recently, Fourier Transform InfraRed (FTIR) absorption spectroscopy has been proposed for glyoxal measurements.^17^

Further methods for glyoxal measurement include derivatisation techniques with a reagent such as *o*‐(2,3,4,5,6‐pentafluorobenzyl)‐hydroxylamine (PFBHA),^18^, ^19^ 2,4‐dinitrophenylhydrazine (DNPH)^20^ and pentafluorophenylhydrazine (PFPH)^21^ with subsequent analysis using Gas Chromatography Mass Spectrometry (GC‐MS) or High Performance Liquid Chromatography with Ultraviolet detection (HPLC‐UV). Their drawbacks are long sampling times and a complex procedure of derivatisation and solvent extraction/evaporation which limits sample frequency. Recent improvements include Solid Phase Microextraction (SPME)^22^ providing a sensitive technique with detection limits down to 3 pptv for glyoxal and a shorter sampling time as well as microfluidic derivatisation techniques of α‐dicarbonyls.^23^

Proton transfer reaction mass spectrometry (PTR‐MS) is a versatile analytical technique that has been applied worldwide in the measurement of VOCs.^24^, ^25^ Glyoxal has been considered as a potential contributor to mass *m/z* 59 in PTR‐MS systems using low resolution, quadrupole mass selection along with propanal and acetone.^26^ The greater mass resolution provided by the PTR‐TOF‐MS means that glyoxal should be measureable distinct from acetone and its isomer propanal. Online, sensitive measurements of glyoxal from PTR‐MS have the potential to provide important results from an independent technique. However, a recent study describing the intercomparison of most of these aforementioned techniques reported that no glyoxal could be observed by PTR‐TOF‐MS up to a concentration of 32 ppbv.^27^ Here, we present a characterization and the calibration of glyoxal measurement with a PTR‐TOF‐MS in detail. First, glyoxal was synthesized and the absence of formaldehyde in the sample confirmed using an UV absorption spectrometer. Then, nitrogen gas was passed over the glyoxal source and the concentration in the ensuing gas stream determined in parallel by UV spectrometry and by PTR‐TOF‐MS. By varying concentration, humidity and monitoring all masses the sensitivity, water dependence and extent of fragmentation in glyoxal measurement by PTR‐TOF‐MS was assessed.

## Material and methods

### Preparation of glyoxal

Monomeric glyoxal was prepared by pyrolysis of glyoxal trimer dihydrate (Sigma‐Aldrich, Germany). A mixture of 1.5‐g trimeric glyoxal and 2.0 g of di‐phosphorous pentoxide (VWR Chemicals, Germany) was heated to 80 °C for 15 min in a vacuum glass line. Subsequently, the mixture was heated to 200 °C, and the evolving gaseous products were collected in a cold trap using a dry ice‐ethanol (−72 °C) bath. In order to remove volatile by‐products, the trap was evacuated for 15 min prior to use. The product was isolated in the form of yellow crystals and was stored for 2 days in a dry ice‐ethanol trap under nitrogen.

### Instrumentation

**Figure 1:**
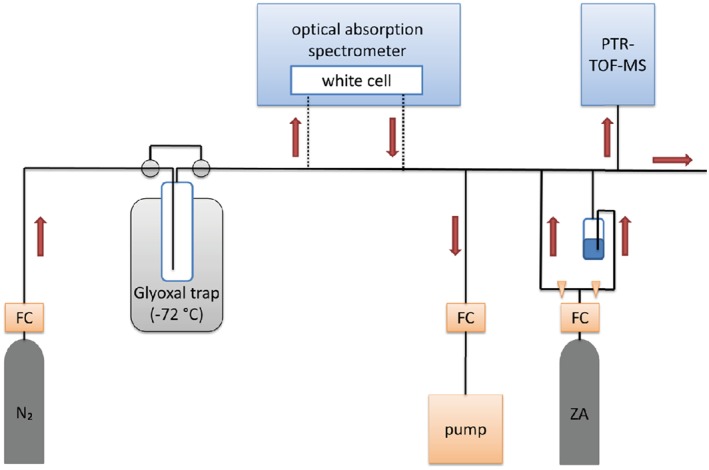
Experimental setup for the calibration of glyoxal. In the upper part of the figure are the UV absorption spectrometer and the Proton Transfer Reaction Time of Flight Mass spectrometer (PTR‐TOF‐MS). The lower part shows the glyoxal source in a dry ice‐ethanol bath at −72 °C, the pump with a flow controller (FC) to reduce the sample flow and the dilution setup with a zero air gas bottle (ZA) and two tubes in order to adjust the humidity with a bubbler. The red arrows represent the flow direction.

A stable concentration of glyoxal in air was produced by passing a steady flow of 500 sccm nitrogen (purity ≥ 99.999 vol%) through the trap containing solid glyoxal at atmospheric pressure and at −72 °C. The concentration of glyoxal eluting from the trap was quantified using UV absorption spectroscopy, see Section on UV Absorption. The sample stream out of the UV spectrometer was further diluted with dry zero air before entering the inlet of the PTR‐TOF‐MS. The experimental setup is shown in Fig. 1. Background measurements were performed by closing off the flow through the trap and directing the nitrogen flow to the UV absorption spectrometer.

In a further set of experiments, the PTR‐TOF‐MS signal of glyoxal was monitored as a function of humidity. To achieve this, a flow of zero air was split into two streams, one of which was humidified using a bubbler filled with distilled water. The ratio of these two flows in combination determined the degree of humidity. Thus, the overall dilution flow added to the sample stream was kept constant, while the humidity was changed. A dry sample was measured at the beginning and end of the relative humidity‐calibration steps in order to check that the source remained stable. The PTR‐TOF‐MS background with dry zero air was taken before and after each measuring day; a background with humid air was taken before the first calibration.

### UV absorption

A custom built optical absorption spectrometer was used to analyse a flowing sample of glyoxal in nitrogen. The absorption spectrum between 250 and 520 nm was measured using a Pyrex absorption cell (110 cm long) fitted with multi‐pass optics to provide an optical path length of 880 cm. The collimated output from a deuterium lamp provided analysis light that transversed the absorption cell eight times before being focused onto the entrance slit of a 0.5 monochromator (B&M Spektronik BM50, equipped with a 300 lines/mm grating blazed at 300 nm) and dispersed onto a diode‐array detector (Oriel INSTAspec 2). The optical density because of glyoxal in the 400 to 460‐nm window was converted to a concentration using a literature reference spectrum.^28^ Upper limits to the HCHO impurity were derived using the spectrum of Bogumil *et al.* 29 scaled as described in the Mainz spectral atlas.^30^

### PTR‐TOF‐MS

The PTR‐TOF‐MS (PTR‐TOF‐MS‐8000, Ionicon Analytik GmbH, Innsbruck, Austria) uses hydroxonium (H_3_O^+^) ions to ionize the analyte molecules. The relatively soft chemical ionization technique results in only weak fragmentation of the analyte. After protonation of the analyte, the resultant ions are focused through a lens system and are directed to the flight tube, where the ions are separated according to their flight time. The travel time before the ions reach the detector, a multi‐channel plate, depends on the mass to charge ratio.^24^, ^31^ To be protonated, the proton affinity of a molecule needs to be greater than the proton affinity of water. In the case of glyoxal, the calculated proton affinity ranges between 161.41 and 165.06 kcal/mol^32^ and is thus only slightly higher than the proton affinity of water, 164.80 kcal/mol. This can complicate its measurement as has been shown previously of formaldehyde.^33^, ^34^

The mass resolution (*m*/∆*m*) of 4000 for the PTR‐TOF‐MS is sufficient to separate protonated glyoxal (59.0128 *m*/*z*) and acetone (59.0491 *m*/*z*), which have the same nominal mass.^35^ This is not possible with a quadrupole PTR‐MS.

The online measurement of the sample stream was performed by using a commercial proton transfer reaction time of flight mass spectrometer. A flow of 200 sccm was drawn from the main sample stream and passed through a heated polyether ether ketone (PEEK) inlet tube (60 °C) to the PTR‐TOF‐MS drift tube. At each calibration step, the flows were kept constant for 10 min. During the test to investigate the dependency on relative humidity all tubing downstream of the addition of humid zero air to the main sample stream was heated to 50 °C. The proton transfer reaction in the drift tube was performed under the following instrumental conditions: drift temperature 60 °C, drift pressure 2.2 mbar, drift voltage 600 V providing an E/N value of 137 Td (1 Td = 10^−17^ cm^2^V^−1^s^−1^). The time resolution of the measurements was 1 min.

The raw PTR‐TOF‐MS data was evaluated with the PTR‐TOF Data Analyser. Detailed information about the methods implemented in this toolbox is summarized elsewhere.^36^ The raw values from the Data Analyser are converted into normalized counts per second (ncps) by dividing the counts for each mass through the counts for the protonated water (H_3_^18^O^+^ on the mass 21 *m*/*z*) and the counts for the first water cluster ((H_2_^18^O)H_3_O^+^ on the mass 39 *m*/*z*) converted into the signal counts of their main isotope. Furthermore, the signals are calculated for a standardized pressure (2 mbar) and temperature (25 °C) in the drift tube. For each calibration step, it took at most 10 min for the signal to stabilize. After this, the signal was averaged for about 5 min. In order to establish a criterion for the humidity adjustments, the ratio of the signals from the 39 *m*/*z* ions and 21 *m*/*z* ions was taken (m39/m21 ratio). The relationship between the m39/m21 ratio and the relative humidity is shown in Fig. S1 in the Supporting Information. To calculate the sensitivity *s* of the humidified samples (humid cal.), the sensitivity of the dry calibration (dry cal.) was multiplied with the relative decrease of the signal compared to the dry calibration. Thus, the following equation was applied:

(1) $$s\left(\text{humid}\;cal.\right)\;=s\left(\text{dry}\;cal.\right)\cdot \text{ncps}\left(\text{humid}\;cal.\right)/\text{ncps}\left(\text{dry}\;cal\text{.}\right)$$

The dry calibration signal was interpolated between the dry measurement at the beginning and at the end in order to correct for changes in the output of the glyoxal source.

## Results and discussion

In order to calibrate the PTR‐TOF‐MS for glyoxal, the mixing ratio of glyoxal emitted from the sample trap was measured by optical absorption as described above. Figure S2 of the Supporting Information shows the optical density measured between 400 and 460 nm (red line), the reference spectrum (black line) and fit residuals (blue line). The mixing ratio derived from this procedure was 8.3 · 10^14^ molecule cm^−3^ with an associated statistical error of less than 1%. The overall uncertainty is estimated as ~10% and is related largely to uncertainty in the glyoxal cross sections. From the concentration and the overall pressure and temperature, the mixing ratio in the exhaust flow of the trap was thus determined to be 34.2 ± 3.4 ppm. This flow of 500 sccm was reduced by drawing out 480 sccm and diluting the residual flow with a stream of zero air to a minimum mixing ratio of glyoxal of 43.0 ± 4.4 ppbv. The overall uncertainty of approximately 10% stems mainly from the accuracy of the cross sections of glyoxal (~5%) and from dilution accuracy based on the error of the mass flow controllers (~5%). No evidence was found for the presence of HCHO with an upper limit of 2% of the concentration of glyoxal. This is based on lack of absorption features at ~330 ± 20 nm where HCHO absorbs most strongly.^29^

**Figure 2:**
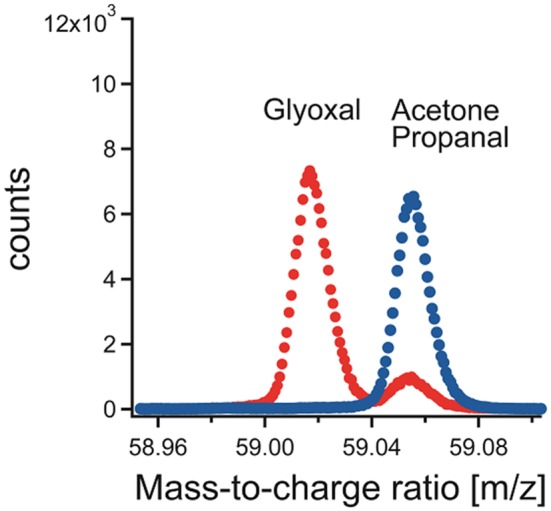
Section of the mass spectrum with protonated masses of glyoxal and acetone/propanal. The red points display the integrated counts during the calibration, and the blue points were recorded during an ambient air measurement.

**Figure 3:**
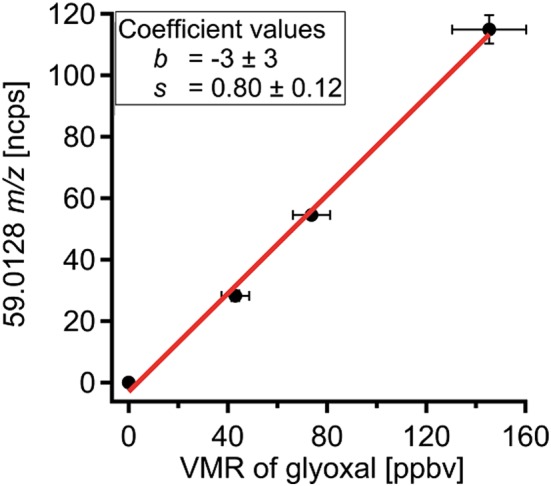
Calibration curve of glyoxal with a dry dilution flow at a m39/m21 ratio of 0.02. The horizontal error bars represent the uncertainty in the concentration of glyoxal and the accuracy of the dilution factor. The vertical error bars stem from the standard deviation of the measured signal.

The signal of protonated glyoxal besides protonated acetone can be seen in Fig. 2. The two peaks are well separated. The red points display the mass spectrum obtained during the calibration of glyoxal, and the blue points show the mass spectrum during the measurement of ambient air. The y‐axis shows the accumulated counts for 1‐h integration time. From the dilution factors, the mixing ratio of glyoxal could be calculated and the corresponding signal of the PTR‐TOF‐MS is displayed in Fig. 3. The sensitivity *s* (at 59.0128 *m*/*z*) was 0.80 ± 0.12 ncps/ppbv at a m39/m21 ratio of 0.02. For comparison, the typical sensitivity for acetone is 25 ncps/ppbv over an order of magnitude higher.

### Humidity calibration

**Figure 4:**
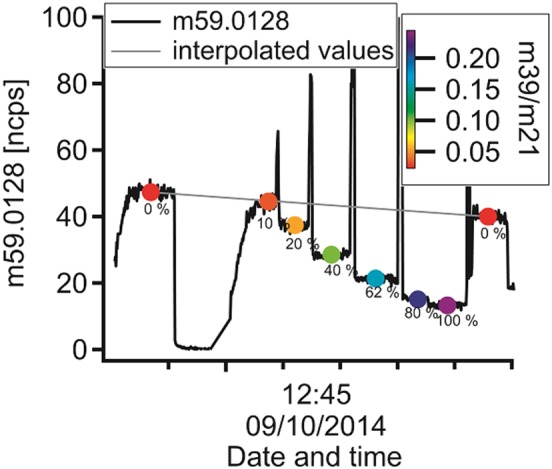
Variation in glyoxal signal during the humid calibration (black solid line). The gray line indicates the interpolated values for glyoxal if no humid flow would be added. The coloured points show the mean values of each step in terms of humidity. The values under each step (in percent) display the proportion of humid flow to the entire dilution flow.

The PTR‐TOF‐MS signal of the humidity dependence is shown in Fig. 4. The black line displays the behaviour of the glyoxal signal. The numbers (in %) under the black line are the amount of humidified air added to the dry dilution flow starting and ending with a dry calibration step. Finally, the coloured dots represent the mean values for each calibration step, and the colour indicates the m39/m21 ratio from a complete dry dilution flow (red) to a complete humid dilution flow (purple).

**Figure 5:**
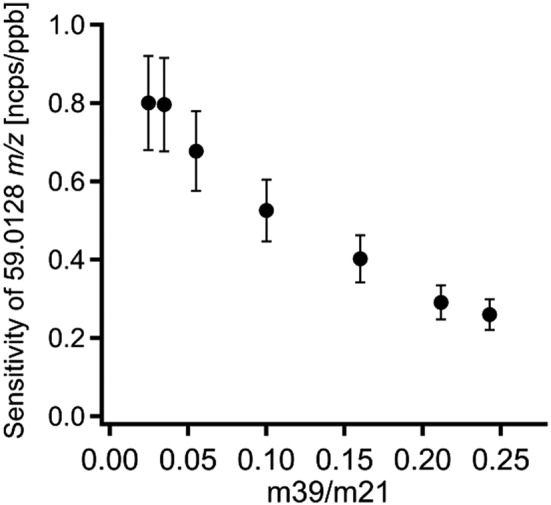
Dependence of the sensitivity on the m39/m21 ratio.

Regarding the PTR‐TOF‐MS data, the second dry sample measurement shows slightly lower values than the first one. From the relative signal of the humid calibration steps to the interpolated signal of the dry calibration step, the sensitivity can be calculated for each calibration step, see Eqn (1). The results are shown in Fig. 5. The sensitivity decreases with increasing humidity. The sensitivities of the calibrations with smallest amount of humidified air (0% and 10% of the dry dilution flow was humidified) lie close to each other. This could possibly be because of an error in the interpolation between the two points of the dry calibration, which is assumed to be a straight line.

The sensitivity ranges between 0.80 ± 0.12 ncps/ppbv for the dry calibration and 0.26 ± 0.04 ncps/ppbv for the humid calibration. The detection limit ranges from 250 pptv for the calibration with the dry dilution flow up to 700 pptv for the calibration with the greatest humidity for 10‐s integration time. The overall uncertainty in the quantification of glyoxal with a PTR‐TOF‐MS was calculated by combining the instrumental precision with systematic errors including the uncertainty of the flow measurements, the accuracy of determining the glyoxal concentration and the error of the calibration. This resulted in an overall uncertainty of ~15%, which is dominated by uncertainty in the glyoxal concentration. The detection limit was determined in zero air without any nearby signals of acetone or propanal and is defined as three times the standard deviation of the background measurements.

The calibration indicates that the PTR‐TOF‐MS sensitivity to glyoxal is low and that the detection limit is high. This limits the use of PTR‐TOF‐MS for ambient air glyoxal measurements because the atmospheric abundance of glyoxal in isoprene‐rich regions is typically only in the range between 10 and 100 pptv.^2^

### Fragmentation of glyoxal

**Figure 6:**
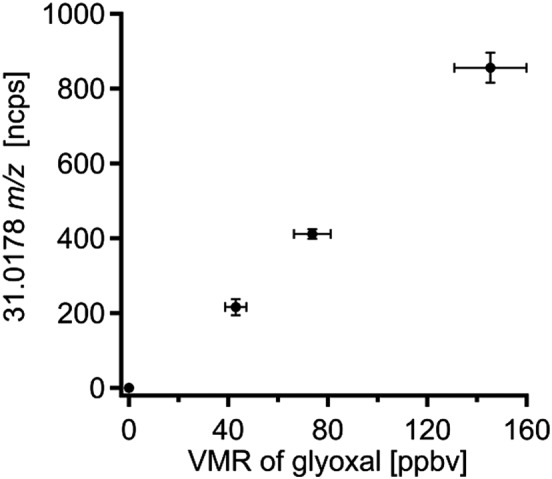
The relationship between the mixing ratio of glyoxal and the measured signal on 31.0178 *m/z* is shown. The horizontal error bars represent the uncertainty in the concentration of glyoxal and the accuracy of the dilution factor. The vertical error bars stem from the standard deviation of the measured signal.

The signal of protonated glyoxal is accompanied by a signal at 31.0178 *m*/*z*. This signal most likely belongs to protonated formaldehyde (H_2_COH^+^). Because there is no evidence that formaldehyde is abundant in the sample flow, it must be formed in the drift tube via the fragmentation of protonated glyoxal. The linear relationship between the signal of 31.0178 *m*/*z* and the supplied mixing ratio of glyoxal is displayed in Fig. 6.

**Figure 7:**
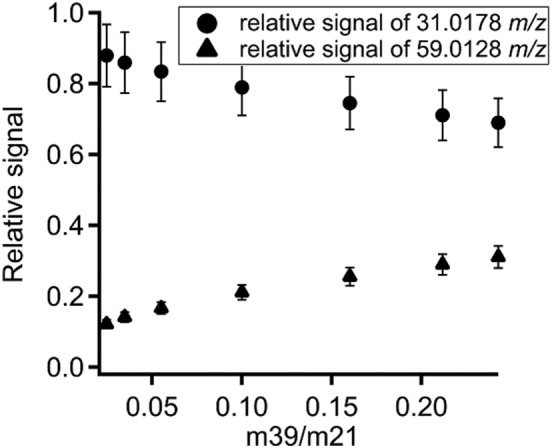
Relative signals of formaldehyde 31.0178 *m/z* (circles) and glyoxal 59.0128 *m/z* (triangles) at different m39/m21 ratios. The relative signals were calculated by dividing the signal of each mass through the sum of the signals of both masses.

**Figure 8:**
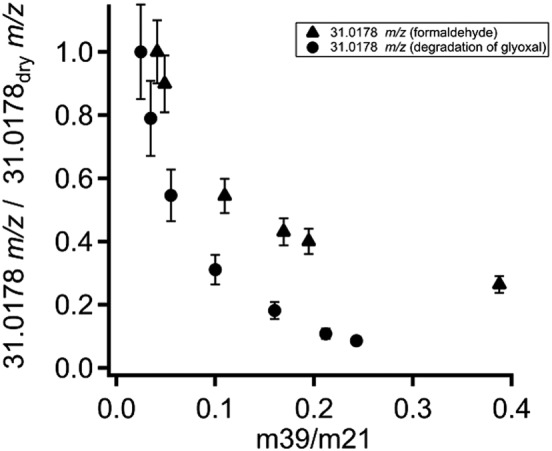
Relative signals of 31.0178 *m/z* obtained from the calibration of glyoxal (circles) and the calibration of formaldehyde (triangles) against relative humidity as expressed by the m39/m21 ratio.

The decreasing sensitivity for glyoxal as well as formaldehyde with increasing humidity is likely caused by the low proton affinity and the relatively high rate of the back reaction (deprotonization). An equivalent increase of the hydrates of both species could not be observed. Figure 7 displays the relative signal of formaldehyde (circles) and glyoxal (triangles), which were calculated from each species divided by the sum of both molecules. It can be seen that the relative signal of formaldehyde is considerably larger than the signal of glyoxal. Altogether the relative signal of glyoxal increases with increasing humidity but the absolute signal exponentially decays as can be seen in Fig. 5. The comparison between the normalized response of the signal 31.0178 *m*/*z* measured during the calibration of glyoxal (circles) and an ordinary calibration of formaldehyde (triangles) at different m39/m21 ratios is shown in Fig. 8. The values were normalized to the signal 31.0178 *m*/*z* of the driest condition. The humidity dependence of the signal 31.0178 *m*/*z* obtained from the calibration of glyoxal is steeper than the curve from the calibration of formaldehyde. This different behaviour could indicate the direct formation of protonated formaldehyde from the fragmentation of protonated glyoxal.

## Conclusion

A PTR‐TOF‐MS was calibrated for gaseous glyoxal. It was demonstrated that the sensitivity declined with increasing humidity, and that the protonated glyoxal fragments to 90% onto the signal of protonated formaldehyde (31.0178 *m*/*z*). The sensitivity and detection limit for dry conditions lie at 0.80 ± 0.14 ncps/ppbv and 250 pptv, respectively, and 0.26 ± 0.07 ncps/ppbv and 700 pptv for humid conditions. The sensitivity is insufficient to measure glyoxal in ambient air using currently PTR‐TOF‐MS technology because typical mixing ratios range between 10 and 100 pptv. Future improvements in sensitivity may, however, make ambient glyoxal measurements accessible.

Two recent studies conducted at the EUPHORE chamber intercompared measurements of glyoxal and methyl glyoxal with different devices and measurement techniques. Both studies concluded that it was not possible to observe glyoxal. Thalman *et al.* tested concentrations of glyoxal up to 32 ppbv resulting in no significant signal of glyoxal in the mass spectra.^23^, ^27^ In this study, we explain why difficulties may have arisen. However, in our study, the lowest mixing ratio of glyoxal of 43 ppbv resulted in a significant signal, and there is no evidence why there would not be an observable signal at lower mixing ratios. The decisive point could be that we optimized our setup in terms of short and heated Teflon tubes in order to minimize wall effects because of the ‘sticky’ nature of glyoxal.

Furthermore, this study shows that a cold‐trap PTR‐MS does not measure protonated glyoxal sensitively at the mass 59 *m*/*z*. However, the signal on mass 31 *m*/*z* should be regarded with caution because molecules like glyoxal and possibly other oxygenated trace gases^37^, ^38^, ^39^ fragment to ions interfering with that mass.

## Supporting information

Figure S1. Relationship between the m39/m21 ratio and the relative humidity in %.
Figure S2 Optical density of glyoxal measured between 400 and 460 nm (red line), the reference spectrum (black line) and fit residuals (blue line).

Supporting info item
