# Supplementary material for: Glyoxal measurement with a proton transfer reaction time of flight mass spectrometer (PTR‐TOF‐MS): characterization and calibration
Source: J Mass Spectrom. 2016 Nov 25;52(1):30–5. doi: 10.1002/jms.3893 (PMC6681138; doi:10.1002/jms.3893)
Supplement: Supplementary file 1 — Supporting info item [file JMS-52-30-s001.pdf]

Figure S1. Relationship between the m39/m21-ratio and the relative humidity in %.

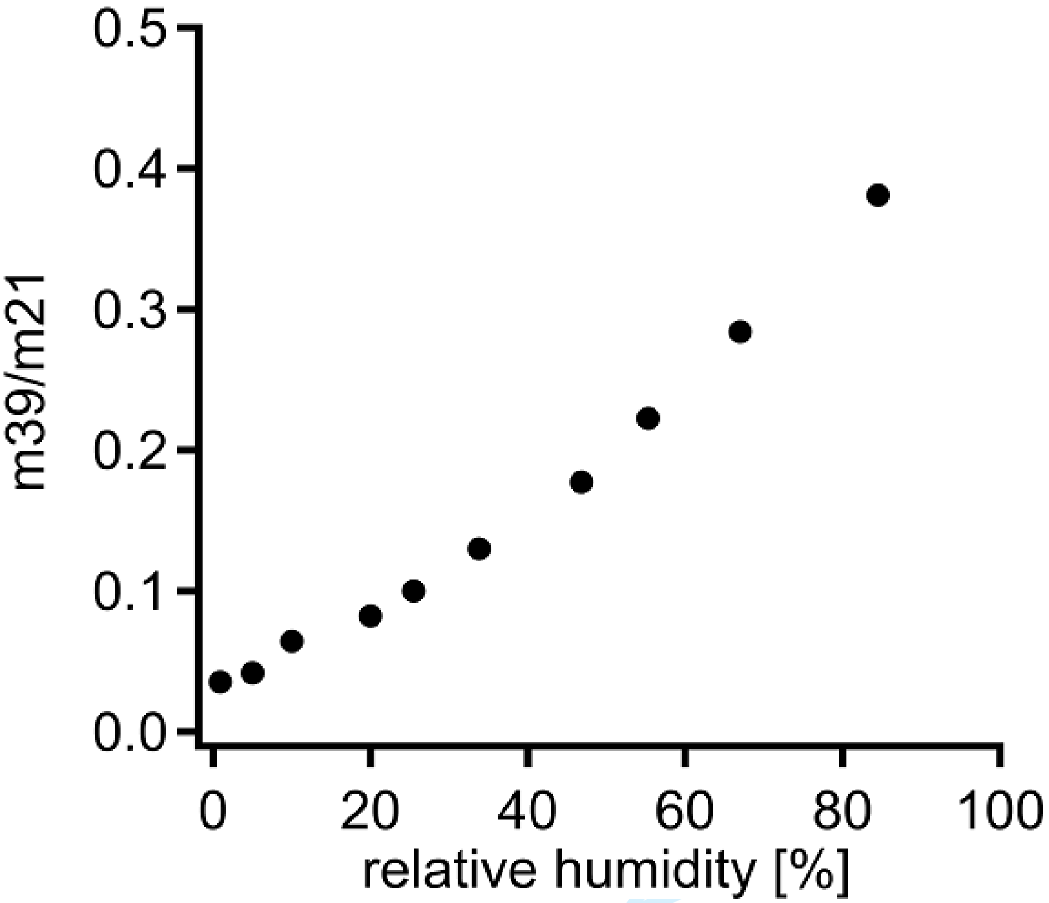

Figure S2 Optical density of glyoxal measured between 400 and 460 nm (red line), the reference spectrum (black line) and fit residuals (blue line).

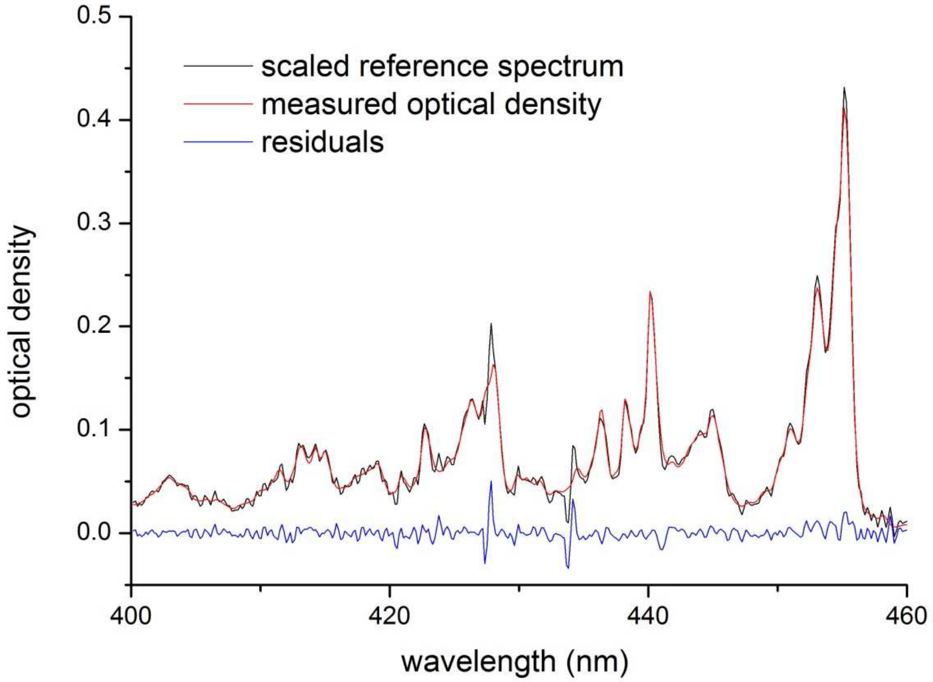

ew Only
